# Aquaporins and Glia

**DOI:** 10.2174/157015910791233178

**Published:** 2010-06

**Authors:** Roberta Albertini, Rossella Bianchi

**Affiliations:** 1 Division of Human Anatomy, Department of Biomedical Sciences and Biotechnologies, University of Brescia, V.le Europa 11, 25123 Brescia, Italy; 2 Department of Human Morphology, University of Milan, Milan, Italy

**Keywords:** Aquaporins, glial cells.

## Abstract

Glial cells coordinate the differentiation, metabolism, and excitability of neurons; they modulate synaptic transmission and integrate signals emanating from neurons and other glial cells. Several evidences underlying the relation between these pathways and the regulatory mechanisms of ion concentration, supporting the role of Aquaporins (AQPs) in these processes.

The goal of this review is to summarize the localization of different isoforms of AQPs in relation to glial cells both in central and peripheral nervous system, underlying AQP involvement in physiological and in pathophysiological conditions such as brain edema, glioma and epilepsy.

## 1. GENERAL CONCEPTS

Maintenance of a stable internal osmotic environment is essential for normal cerebral activity [85].

Astrocytes, that are the most numerous glial cell type and account for one third of brain mass [35], are involved in the maintenance of the blood–brain barrier (BBB), in the regulation of water and ion homeostasis and aminoacid neurotransmitter metabolism, as well as energy and nutrient support of neurons. Of particular importance is regulatory volume decrease that allows cells to remove excess water from their cytoplasm in attempt to maintain a proper water balance. In particular water extrusion occurs through specialized water channels called Aquaporins (AQPs) [74], although in some cells water permeates lipid membranes directly [109] or passes the membrane through unrelated channels [27].

The expression of six isoforms of AQP protein (AQP1, 3, 4, 5, 8, 9) has been reported in the glial cells Table **1**, being identified in astrocytes (AQP1, 3, 4, 5, 8, 9), oligodendrocytes (AQP8), tanycytes (AQP9) and ependymal cells (AQP1, 4, 9) [5, 40, 49, 52, 58, 63, 93]. Moreover a recent study demonstrated the expression of AQP4 mRNA and protein within reactive microglial cells *in vivo* [95].

Although numerous studies have reported AQP localization and function in the central nervous system (CNS), little is known about the expression and function of AQPs in the peripheral nervous system (PNS). In particular, there has been controversy over the localization of AQP1 in the PNS. Nevertheless, Gao and co-workers [28] reported AQP1 expression in glial elements while more recent studies have demonstrated the presence of neural elements positive for AQP1 in the enteric nervous system [54]. Moreover AQP1 was found in the PNS of dorsal root, trigeminal and nodose ganglia [57, 67, 84]. AQP2 expression was recently reported also in Schwann cells of dorsal root and trigeminal ganglia during pain condition [12, 13].

These findings suggest that members of the AQP family are differentially expressed in the peripheral versus CNS.

## 2. AQUAPORINS AND GLIAL CELLS

### 2.1 AQP1

AQP1 mediates water transport in several organs where it plays an important role, such as the formation of urine and aqueous humor in the eye [97, 110] or cerebrospinal fluid (CSF) in the brain. Brain AQP1 is mainly expressed in the CSF-facing membranes of the ventricular choroid plexus, where it regulates the formation of CSF, partly because of its ion channel function [66, 87].

Increasing evidences indicate that brain astrocytes express AQP1 under pathologic conditions. AQP1 is found in reactive astrocytes accumulating in the lesions of subarachnoid hemorrhage [3], contusion [92], Creutzfeldt-Jacob disease [79], cerebral infarction and multiple sclerosis [82]. Moreover, a recent study showed that the expression of this protein is increased in cortical astrocytes at the early stage of Alzheimer disease, suggesting a pathological role of abnormal regulation of water transport in this condition [73].

After spinal cord injury (SCI) there was evidence that reactive astrocytes show a significant increase of AQP1 expression surrounding the lesion site, while reactive astrocytes distant from the lesion express markedly less it [58]. So, given that hypoxia stimulates astrocytic migration [106] it is possible that hypoxic conditions after SCI trigger AQP1 synthesis in astrocytes, as an attempt of injured spinal cords to facilitate astrocytic migration to the lesion site. A role for AQPs in facilitating cell migration was first reported for AQP1 and vascular endothelial cells in mice implanted with melanoma tumours [81]. Nevertheless, molecular mechanisms responsible for AQP involvement in cell migration are unknown. Two theories have recently been suggested [71]: according to the first theory actin depolymerisation and ion influx at the leading edge of migrating cells increase local cytoplasm osmolarity, thus promoting water influx through the cell membrane, facilitated by AQPs, causing a local rise in hydrostatic pressure that expands the cell membrane. Then, actin repolymerisation stabilizes the membrane protrusion. According to the second theory, migrating cells undergo rapid changes in cell shape and cell volume as they move through the irregularly shaped brain extracellular space (ECS) between stationary cells. AQPs accelerate cell migration by facilitating the transmembrane water fluxes that mediate such cell volume changes. AQP1 is expressed also in the ependymal cells of spinal cord around the central canal and more robustly in the sensory fibers of the superficial laminae of the dorsal horn [84]. Its levels was significantly and persistently elevated for up to 11 months after SCI in sensory axons, neurons but also in astrocytes and ependymal cells, despite considerable loss of neurons and axons at the site of injury [58]. Hypoxic conditions may contribute to chronic accumulation of water within neurons and cytotoxic edema that can account for the increased water content in chronically injured spinal cords, given that the antioxidant melatonin significantly decreases AQP1 expression induced by SCI [59]. Sustained up-regulation of AQP1 in ependymal cells may result in the over-production of CSF and formation of CSF-filled cysts after SCI, a serious and untreatable complication of SCI [23]. In fact, it has already been shown that AQP1 has a role in the formation of CSF-filled cysts [7, 44].

Moreover, AQP1 may play during the intake of water that precedes axonal elongation. Nesic *et al*. [58] demonstrated that the pattern of AQP1 expression in the dorsal horns was similar to that of GAP-43, important in axonal growth during embryogenesis, in synaptic remodeling and plasticity and in regeneration after injury [15, 36]. GAP-43 that resides in the growth cones of growing neuritis where it interacts with F-actin-associated adhesion molecules and/or extracellular matrix complexes to promote neurite extension [31], co-localized with AQP1 in both uninjured and injured spinal cords. AQP1 and GAP-43 labeling were found in particular within unmyelinated, small-diameter nerve fibers in the superficial laminae of the dorsal horns; this indicates a high degree of plasticity of sensory axons. Therefore AQP1 expression in spinal cord may have a role in axonal remodeling and plasticity, necessary for normal sensory processing.

In brain tumours AQP1 expression increases with the grade of malignancy [64, 81]. In glioblastoma multiformes, in addition to vascular alterations, there are important metabolic changes compared to normal brain tissue. In fact, glioma cells can engage in high rates of aerobic glycolysis, resulting in increased glucose consumption and production of lactic acid even under normoxia [45]. The increase in lactic acid production and subsequent acidification of extracellular space likely contribute to the invasive potential of cancer cells [29]. The ability of cells to transport excess H^+^ from intracellular to extracellular space may also require movement of H_2_O in the same direction [34], suggesting another potential function for AQP1 in brain tumours, besides to contribute to vasogenic edema formation.

In the PNS satellite cells of the trigeminal ganglia of rat show AQP1 expression [57] (Fig. (**1**)). Glial Fibrillary Acidic Protein (GFAP)-positive Schwann cells are known to express AQP1 too [28, 57] (Fig. (**1**)) but high resolution studies using electron microscopy are needed to determine the nature of AQP1-positive cells in nerve bundles and the detailed subcellular localization of AQP1 in both nerve plexuses and bundles in the PNS.

### 2.2 AQP2

Many studies suggest that AQP2 is exclusively expressed in the renal collecting duct [37] but there is increasing of evidence that AQP2 is also expressed in several extra-renal locations such as the male reproductive tract [89], the inner ear [51], pancreatic islets, selected cells in gastric pits, intestinal and colonic epithelium and fallopian tubes [89].

Mobasheri and co-workers [53] reported that in the CNS AQP2 was found also in selected regions of the CNS including ependymal cell layer, spinal cord, subcortical white matter and hippocampus.

Moreover Borsani *et al*. [12] in a murine inflammatory pain model showed for the first time that AQP2 is expressed also in Schwann and satellite cells of trigeminal ganglia, while in normal condition it is not found. Moreover, after chronic constriction of sciatic nerve (CCI) in rat, AQP2 expression increased in small-diameter dorsal root ganglia neurons but also in Schwann cells [13]. On the basis of these data AQP2 may be involved in pain transmission in the PNS. In fact, many studies have provided indirect evidence of the contribution of osmosis in the pain pathway. Moreover, AQP2 could be involved in cellular processes characterized by ion influx like transduction of signal injury, nerve conduction and synaptic transmission.

### 2.3 AQP3

Unique evidence of AQP3 expression in glial cells was showed from Yamamoto *et al*. [108]. In fact, through RNase protection assay and reverse transcription-polymerase reaction (RT-PCR) they found AQP3 expression in astrocytes as well as in neurons.

### 2.4 AQP4

A general paradigm is that AQP4 is not expressed in excitable cells, but is found in supporting cells as astrocytes and ependyma in the nervous system and Muller glia in the retina. In fact, it is strongly expressed at the borders between brain parenchyma and major fluid compartments including astrocytes foot processes (brain-blood), glia limitans (brain-subarachnoid CSF), as well as ependymal cells and subependymal astrocytes (brain-ventricular CSF) [63, 78].

The osmosensitive organs display a unique pattern of AQP4 compartmentation [63]. In these organs the astrocytes form lamellae that contain high concentrations of AQP4, also at sites that do not face the brain surface or capillaries. Although AQP4 expression is polarized in astrocytes foot processes adjacent to endothelial cells, in the absence of endothelia (e.g. cultured astrocytes and malignant astrocytes) [78, 80, 105] AQP4 redistributes throughout the astrocyte cell membrane, suggesting that endothelial cells signal astrocytes to polarize AQP4 expression in the cell membrane [93].

Recent study using AQP4 knock-out mice indicated that AQP4 is involved in the formation and resolution of brain and spinal cord edemas [47, 69, 70]. Compared to normal mice, AQP4 knock-out mice exhibit reduced brain edema and neurologic improvement following ischemic brain injury [46, 99]; in these animals the rate of osmotic swelling is significantly reduced in the deeper lamina of the dorsal horn of the spinal cord [86]. In fact it may favor high transmembrane fluxes of water with minimal osmotic perturbation [6].

In general AQP4 expression is up-regulated in astrocytes associated with brain edema. For exemple, AQP4 over-expression in human astrocytomas correlates with the presence of brain edema on magnetic resonance scans [80]. Correlations between AQP4 expression and brain edema have been reported after brain ischaemia [17, 50], and traumatic brain injury in rodents [17, 91]. Moreover, in glioblastoma it was recently demonstrated an up-regulation and redistribution of AQP4 accompanied by a loss of its polarized expression pattern and so the evidence for a role of it in vasogenic edema formation [80, 102, 104].

AQP4 also facilitates the elimination of excess brain water. Excess water is eliminated primarily through the glia limiting membranes into the CSF. Greater brain water accumulation and intracranial pressure were found in AQP4 knock-out versus wild-type mice with brain tumour, brain abscess, focal cortical-freeze injury, and after infusion of normal saline directly into brain extracellular space [11, 69, 70], indicating that vasogenic edema fluid is eliminated by an AQP4-dependent route. Also, in a kaolin injection model of obstructive hydrocephalus, AQP4 knock-out mice develop more marked hydrocephalus than wild-type mice [10], probably due to reduced water clearance through the ependymal and BBB. Moreover, animal models imply a protective effect of AQP4 by facilitating excess fluid assembled in the brain parenchyma after BBB disruption [69, 70].

Changes in astrocyte shape and function are known also to occur in association with human immunodeficiency virus (HIV) dementia (HIVD). Using Western Blot analysis, Hillaire and collaborators [88] showed that immunoreactivity for AQP4 is elevated in brain homogenates from the mid frontal gyrus of patients who died with HIVD. Of interest, a significant increase is observed in homogenates from HIV-infected individuals without dementia too. Nevertheless, additional studies are necessary to determine whether altered AQP4 expression represents a protective and/or maladaptive response to CNS inflammation.

Recent studies have found also changes in astroglia Kir channels and AQP4 water channels in temporal lobe epilepsy specimens [9]. Alterations in astroglial water regulation could, in fact, powerfully affect excitability. Brain tissue excitability is sensitive to osmolarity and size of the ECS [83]. Decreasing ECS volume produces hyperexcitability and enhanced epileptiform activity [16, 19, 68], while increasing ECS volume with hyperosmolar medium attenuates epileptiform activity [19, 68]. Several lines of evidence support the hypothesis that AQP4 and Kir4.1 may act in concert in K^+^ and H_2_O regulation [85]. In fact, K^+^ re-uptake into glial cells could be AQP4-dependent, as water influx coupled to K^+ ^influx is thought to underlie activity-induced glial cell swelling [100, 101]. In addition studies in the retina have demonstrated subcellular co-localization of AQP4 and Kir4.1 *via *both electron microscopic and co-immunoprecipitation analyses [18, 55] while Kir4.1^-/-^ mice showed impaired retinal and cochlear physiology presumably due to altered K^+^ metabolism [42, 43, 48]. Moreover, afferent stimulation of hippocampal slices from α-syntrophin-deficient mice demonstrates a deficit in extracellular K^+^ clearance [2]. These data are consistent with the idea that AQP4 and Kir4.1 participate in clearance of K^+^ following neural activity. Eid and collaborates [21] reported that the loss of perivascular AQP4 in mesial temporal lobe of patients with epilepsy results in a perturbed flux of water through astrocytes leading to an impaired buffering of extracellular K^+^ and an increased propensity for seizure. Nevertheless, another study reported that a significant increase in AQP4 is observed in sclerotic, but not in non-sclerotic, hippocampi obtained from patients with medically intractable temporal lobe epilepsy [39]. However, further studies are required to clarify the expression and functional interaction of AQP4 and Kir4.1 in the hippocampus and their changes during epileptogenesis.

AQP4 seems to be involved also in autism. In fact, while earlier studies did not show evidence of astrogliosis or microglial activation [8], more recent works have suggested a role for neuroglial activation in autism [38, 72]. Several recent reports have showed evidence for astroglial activation in cerebellum, frontal, and cingulate cortex [25, 38, 98], adding to the weight of pathologic evidence in favor of immune dysregulation in brains of autistic subjects. 

Pardo and co-workers [72] suggested that, while it is unclear how and when microglia and astroglia become activated, it may result from intrinsic disturbances in neuroglial function or neuronal-neuroglial interactions during brain development or from extrinsic effects resulting from unknown factors that disturb prenatal or postnatal development. A role for neuroglial activation in autism is suggested by altered expression of GFAP, a marker of astroglial in brain [1, 98] and CSF [1] of subjects with autism. In addition to GFAP, altered expression of AQP4 has been associated with this neurologic disease; in particular AQP4 expression was decreased in cerebellum of subjects with autism. Prenatal influence infection in mice results in a down-regulation of AQP4 expression in the offspring at birth [26] that persists at adolescence [24]. Decreased AQP4 expression may mean that cell structure, cell volume and ionic homeostatis are compromised. Nicchia and co-workers [62] showed that cultured astrocytes from AQP4 knock-out mice had altered morphology and reduced osmotic permeability. These changes suggest abnormal glial-neuronal communication in brains of subjects with autism.

AQP4 is abundantly expressed in the basolateral membrane of the ependyma and glia limitans lining subependymal layer and pia. This distribution feature indicates that AQP4 provides a highly efficient pathway to convey the redundant water from parenchyma to ventricle system and subarachnoid space [3, 30, 63]. The highly polarized expression of AQP4 suggests it may be involved in maintaining the structural and functional integrity of the ependyma. Immunohistochemistry and electromicroscopy demonstrated that AQP4 deletion resulted in decreased expression of gap junction protein connexin43 (Cx43) [41], that is the main gap-junction protein in astrocytes as well as ependymal cells [77].

Tomas-Camardiel and colleagues [95] provided for the first time direct evidence demonstrating the expression of AQP4 mRNA and protein within reactive microglial cells *in vivo*. In fact the intranigral injection of lipopolysaccharide, that mimics the BBB disruption in memingoencephalitis [75] and provokes severe vasogenic brain edema, produced an area of disappearance of astrocytes probably due to the opening of the BBB which filled with reactive microglia expressing AQP4 mRNA and protein. With no exception, all vimentin-labelled in the astrocytes depleted area exhibited a pseudopodic to globular-shaped morphology, typical of reactive microglia [76, 90]. It is possible that all cells expressing AQP4 expression within microglial cells may represent a molecular adaptation to maintain ion water homeostasis in the injured brain. The loss of astrocytes and proliferation of activated microglia could suggest that activated microglia is implicated in the clearance of potassium ions (K^+^) and restoration of osmotic equilibrium in absence of astrocytes. It is well known, in fact, that glial cells play an important role in regulating the homeostasis to ensure an appropriate neuronal environment. The astrocytes may help clear excess K^+^ around active neuron [60] and in this context AQP4 seems to play an essential role [56].

### 2.5 AQP5

In the CNS AQP5 was detected in astrocytes and in neurons [108]. In primary cultures of rat astrocytes, the treatment with dibutyryladenosine 3’, 5’-cyclic monophosphate (dbcAMP), an protein kinase A (PKA) activator, caused decreases in AQP5 mRNA and protein in time- and concentration-dependent manners [107]. Moreover, the effects of dbcAMP on the cells were inhibited by pretreatment with PKA inhibitor suggesting that AQP5 is regulated from PKA [107], one of the major signal transduction pathways in astrocytes regulating cell growth, mRNA expression and enzyme activation [4, 20]. Interestingly, AQP5 showed a transient up-regulation (about 3-fold) and subsequent down-regulation of its expression within 20h of reoxygenation after hypoxia [108]. This result suggests that this protein may be one of the possible candidates for inducing the intracranial edema in the CNS after ischemia injury.

### 2.6 AQP8

AQP8 expression was initially described in the testis, pancreas, placenta and liver [33]. By Northern Blot analysis, AQP8 RNA is not observed in the brain [33]. Nevertheless, AQP8 expression is observed in the spinal cord in particular in the ependymal cells lining the central canal and a faint staining in cells surrounding the canal suggesting a small amount of AQP8 expression in astrocytes too [65]. In a previous study AQP8 was found in astrocytes and in oligodendrocytes using RNase protection assay and the RT-PCR [108]. The expression pattern of AQP8 suggests that this protein may play a role in concert with AQP4 and AQP9 in water transport process. The AQP4 expressed in the perivascular foot processes may participate in transport across the perivascular space, while AQP8 could then facilitate transport into the central canal.

### 2.7 AQP9

AQP9 is a water channel transporting glycerol, mannitol and urea. It was originally identified in human leukocytes [32], is also expressed in liver, testis, and brain [96]. In the brain, AQP9 is expressed in a subset of GFAP-positive ependymal cells lacking cilia, called tanycytes. The tanycytes are found in circumventricular organs of the third ventricle lacking a BBB, such as the mediobasal hypothalamus, subfornical organ, and pineal gland [22, 61]. There are conflicting reports about AQP9 expression in the subset of ciliated ependymal cells [5, 22]. AQP9 is also expressed in astrocytes of the glia limitans and white matter tracts. In contrast to AQP4, which is expressed primarily in the foot-processes of astrocytes, AQP9 is expressed throughout the astrocyte cell bodies and processes in the brain [5]. Like in the brain, AQP9 staining was found in the glia limitans and a subset of cells in the white matter tracts of the spinal cord [65].

AQP9 has been hypothesized to play a role in extracellular water homeostasis and edema formation similar to AQP4 [5] and seems also to facilitate glycerol and monocarboxylate diffusion [14, 96]. As previously described by Badaut and colleagues [5], AQP9 could also play a role in clearing lactate from the extracellular space in pathological ischemic conditions such as stroke and spinal cord injury where lactic acidosis is common. Moreover the AQP9 immunoreactivity was found to be increased at the tumour border, but not within the tumour [103]. Nevertheless, in human glioblastoma most glioma cells throughout the tumour revealed a strong AQP9 expression across the whole surface of the cells. In human astrocytic tumours AQP9 expression were both increased compared with normal brain tissue for all grades of astrocytic tumours and that expression was greater in high-grade tumours than in low-grade ones [94]. The increase of AQP9 expression may counteract the glioma-associated lactic acidosis by clearance of glycerol and lactate from the extracellular space and could be involved in the energy metabolism of the glioma [103].

AQP9 may play an important role in the malignant progression of brain tumours and it can be used as a biomarker for molecular diagnosis and as a new target for gene therapy, but the molecular mechanisms by which AQP9 affects the proliferation and apoptosis of astrocytic tumours need to be studied further.

## CONCLUSIONS

The discovery of AQPs has provided a molecular basis for understanding water transport in a number of tissues including the nervous system. In fact, emerging evidences suggest that brain AQPs play important roles for the dynamic regulation of brain water homeostasis and for the regulation of CSF. A tissue-specific pattern of AQP expression in the nervous system and in different type of glia cells, supports the idea that water movement in brain tissues is carefully regulated in concert with other transport processes from the micro- to macroscopic levels and that AQPs serve as elements of complex signaling assemblies.

Knock-out mice have confirmed the role of AQPs in transepithelial fluid transport, in cell migration, in regulation of glycerol and in forming CSF. Moreover, recent data support roles for AQPs in glial system, both in physiological and in pathophysiological conditions. Glial expression of AQPs is in fact altered in brain edema conditions and in presence of stroke, tumour and infection but also in neurologic and neurodegenerative disease such as epilepsy, HIVD, autism and Alzheimer.

## Figures and Tables

**Fig. (1):**
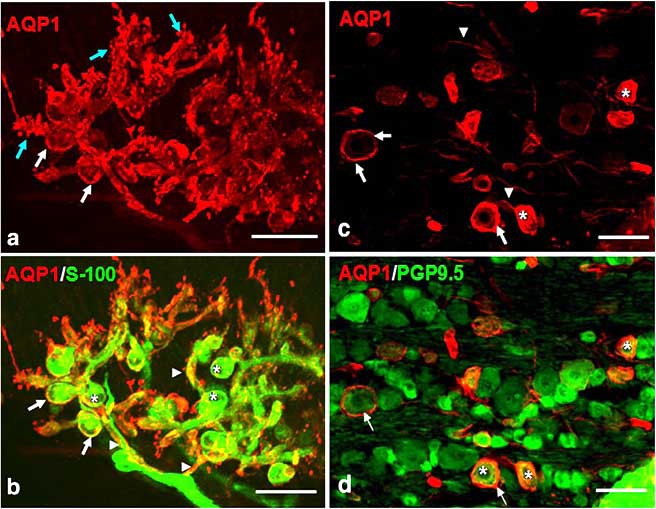
**Immunofluorescent images of the periodontal ligament (a-b) and of the trigeminal ganglia (c-d) of rat.** AQP1 expression (red) in the periodontal Ruffini endings **(a)**, in particular in dendritic axon profiles including microprojections (blue arrows) and round cell bodies of the terminal Schwann cells (white arrows) and in trigeminal ganglia **(c)**, in particular in satellite cells (arrows) and trigeminal neurons (asterisk). Double immunostaining with AQP1 (red) and S-100 protein (green) **(b)**, indicates the co-localization of AQP1 with S-100 protein in the cell membrane of the terminal Schwann cells and their cytoplasmic extensions (arrowheads). The nuclei of the terminal Schwann cells (asterisks) lack AQP1 immunoreaction. Double immunostaining with AQP1 (red) and PGP9.5 (green) **(d)**, indicates the localization of AQP1 in satellite cells while asterisk in trigeminal neurons. Scale bar: 25 µm **(a-b)**; 75 µm **(c-d)**. Figures from Nandasena *et al*. [57], reprinted with permission of Elsevier.

**Table 1:** Localization of Aquaporins in Glial Cells

AQP isoform	Glial localization
AQP1	astrocytes, ependymal cells, Schwann cells, satellite cells
AQP2	ependymal cells, Schwann cells, satellite cells
AQP3	astrocytes
AQP4	astrocytes, ependymal cells, microglial cells
AQP5	astrocytes
AQP8	astrocytes and ependymal cells of spinal cord, oligodendrocytes
AQP9	astrocytes, ependymal cells, tanycytes

Table representing the different isoforms of aquaporin protein in the glial cells of central and peripheral nervous system.

## References

[1] Ahlsén G, Rosengren L, Belfrage M, Palm A, Haglid K, Hamberger A, Gillberg C (1993). Glial fibrillary acidic protein in the cerebrospinal fluid of children with autism and other neuropsychiatric disorders. Biol. Psychiatry.

[2] Amiry-Moghaddam M, Otsuka T, Hurn PD, Traystman RJ, Haug FM, Froehner SC, Adams ME, Neely JD, Agre P, Ottersen OP, Bhardwaj A (2003). An alpha-syntrophin-dependent pool of AQP4 in astroglial end-feet confers bidirectional water flow between blood and brain. Proc. Natl. Acad. Sci. USA.

[3] Amiry-Moghaddam M, Ottersen OP (2003). The molecular basis of water transport in the brain. Nat. Rev. Neurosci.

[4] Arcuri C, Bocchini V, Guerrieri P, Fages C, Tardy M (1995). PKA and PKC activation induces opposite glial fibrillary acidic protein (GFAP) expression and morphology changes in a glioblastoma multiform cell line of clonal origin. J. Neurosci. Res.

[5] Badaut J, Hirt L, Granziera C, Bogousslavsky J, Magistretti PJ, Regli L (2001). Astrocyte-specific expression of aquaporin-9 in mouse brain is increased after transient focal cerebral ischemia. J. Cereb. Blood Flow Metab.

[6] Badaut J, Nehlig A, Verbavatz J, Stoeckel M, Freund-Mercier MJ, Lasbennes F (2000). Hypervascularization in the magnocellular nuclei of the rat hypothalamus: relationship with the distribution of aquaporin-4 and markers of energy metabolism. J. Neuroendocrinol.

[7] Basaldella L, Orvieto E, Dei Tos AP, Della Barbera M, Valente M, Longatti P (2007). Causes of arachnoid cyst development and expansion. Neurosurg. Focus.

[8] Bauman ML, Kemper TL, Bauman ML, Kemper TL (1994). Neuroanatomic observations of the brain in autism. The neurobiology of autism.

[9] Binder DK, Steinhäuser C (2006). Functional changes in astroglial cells in epilepsy. Glia.

[10] Bloch O, Auguste KI, Manley GT, Verkman AS (2006). Accelerated progression of kaolin-induced hydrocephalus in aquaporin-4-deficient mice. J. Cereb. Blood Flow Metab.

[11] Bloch O, Papadopoulos MC, Manley GT, Verkman AS (2005). Aquaporin-4 gene deletion in mice increases focal edema associated with staphylococcal brain abscess. J. Neurochem.

[12] Borsani E, Bernardi S, Albertini R, Rezzani R, Rodella LF (2009). Alterations of AQP2 expression in trigeminal ganglia in a murine inflammation model. Neurosci. Lett.

[13] Buffoli B, Borsani E, Rezzani R, Rodella LF (2009). Chronic constriction injury induces aquaporin-2 expression in the dorsal root ganglia of rats. J. Anat.

[14] Carbrey JM, Gorelick-Feldman DA, Kozono D, Praetorius J, Nielsen S, Agre P (2003). Aquaglyceroporin AQP9: solute permeation and metabolic control of expression in liver. Proc. Natl. Acad. Sci. USA.

[15] Carulli D, Buffo A, Strata P (2004). Reparative mechanisms in the cerebellar cortex. Prog. Neurobiol.

[16] Chebabo SR, Hester MA, Aitken PG, Somjen GG (1995). Hypotonic exposure enhances synaptic transmission and triggers spreading depression in rat hippocampal tissue slices. Brain Res.

[17] Chen CH, Xue R, Zhang J, Li X, Mori S, Bhardwaj A (2007). Effect of osmotherapy with hypertonic saline on regional cerebral edema following experimental stroke: a study utilizing magnetic resonance imaging. Neurocrit. Care.

[18] Connors NC, Adams ME, Froehner SC, Kofuji P (2004). The potassium channel Kir4.1 associates with the dystrophin-glycoprotein complex *via* alpha-syntrophin in glia. J. Biol. Chem.

[19] Dudek FE, Obenhaus A, Tasker JG (1990). Osmolality–induced changes in extracellular volume alter epileptiform bursts independent of chemical synapses in the rat: Importance of non-synaptic mechanisms in hippocampal epileptogenesis. Neurosci. Lett.

[20] Dugan LL, Kim JS, Zhang Y, Bart RD, Sun Y, Holtzman DM, Gutmann DH (1999). Differential effects of cAMP in neurons and astrocytes. Role of B-raf. J. Biol. Chem.

[21] Eid T, Lee TS, Thomas MJ, Amiry-Moghaddam M, Bjørnsen LP, Spencer DD, Agre P, Ottersen OP, de Lanerolle NC (2005). Loss of perivascular aquaporin 4 may underlie deficient water and K^+^ homeostasis in the human epileptogenic hippocampus. Proc. Natl. Acad. Sci. USA.

[22] Elkjaer M, Vajda Z, Nejsum LN, Kwon T, Jensen UB, Amiry-Moghaddam M, Frøkiaer J, Nielsen S (2000). Immunolocalization of AQP9 in liver, epididymis, testis, spleen, and brain. Biochem. Biophys. Res. Commun.

[23] Evans A, Stoodley N, Halpin S (2002). Magnetic resonance imaging of intraspinal cystic lesions: a pictorial review. Curr. Probl. Diagn. Radiol.

[24] Fatemi SH, Folsom TD, Reutiman TJ, Lee S (2008). Expression of astrocytic markers aquaporin 4 and connexin 43 is altered in brains of subjects with autism. Synapse.

[25] Fatemi SH, Laurence J, Araghi-Niknam M, Stary JM, Rizvi S (2002). Glial fibrillary acidic protein is elevated in superior frontal and parietal cortices of autistic subjects. Int. J. Neuropsychopharmacol.

[26] Fatemi SH, Pearce DA, Brooks AI, Sidwell RW (2005). Prenatal viral infection in mouse causes differential expression of genes in brains of mouse progeny: a potential animal model for schizophrenia and autism. Synapse.

[27] Fischbarg J, Kuang KY, Vera JC, Arant S, Silverstein SC, Loike J, Rosen OM (1990). Glucose transporters serve as water channels. Proc. Natl. Acad. Sci. USA.

[28] Gao H, He C, Fang X, Hou X, Feng X, Yang H, Zhao X, Ma T (2006). Localization of aquaporin-1 water channel in glial cells of the human peripheral nervous system. Glia.

[29] Gatenby RA, Gillies RJ (2007). Glycolysis in cancer: a potential target for therapy. Int. J. Biochem. Cell. Biol.

[30] Hasegawa H, Ma T, Skach W, Matthay MA, Verkman AS (1994). Molecular cloning of a mercurial-insensitive water channel expressed in selected water-transporting tissues. J. Biol. Chem.

[31] He Q, Dent EW, Meiri KF (1997). Modulation of actin filament behavior by GAP-43(neuromodulin) is dependent on the phosphorylation status of serine 41, the protein kinase C site. J. Neurosci.

[32] Ishibashi K, Kuwahara M, Gu Y, Tanaka Y, Marumo F, Sasaki S (1998). Cloning and functional expression of a new aquaporin (AQP9) abundantly expressed in the peripheral leukocytes permeable to water and urea, but not to glycerol. Biochem. Biophys. Res. Commun.

[33] Ishibashi K, Kuwahara M, Kageyama Y, Tohsaka A, Marumo F, Sasaki S (1997). Cloning and functional expression of a second new aquaporin abundantly expressed in testis. Biochem. Biophys. Res. Commun.

[34] Ivanov S, Liao SY, Ivanova A, Danilkovitch-Miagkova A, Tarasova N, Weirich G, Merrill MJ, Proescholdt MA, Oldfield EH, Lee J, Zavada J, Waheed A, Sly W, Lerman MI, Stanbridge EJ (2001). Expression of hypoxia-inducible cell-surface transmembrane carbonic anhydrases in human cancer. Am. J. Pathol.

[35] Kandel ER, Kandel ER, Schwartz JH, Jessel TM (1991). Nerve cell and behavior. Principles of neural science.

[36] Kruger L, Bendotti C, Rivolta R, Samanin R (1993). Distribution of GAP-43 mRNA in the adult rat brain. J. Comp. Neurol.

[37] Kwon TH, Hager H, Nejsum LN, Andersen ML, Frøkiaer J, Nielsen S (2001). Physiology and pathophysiology of renal aquaporins. Semin. Nephrol.

[38] Laurence JA, Fatemi SH (2005). Glial fibrillary acidic protein is elevated in superior frontal, parietal and cerebellar cortices of autistic subjects. Cerebellum.

[39] Lee TS, Eid T, Mane S, Kim JH, Spencer DD, Ottersen OP, de Lanerolle NC (2004). Aquaporin-4 is increased in the sclerotic hippocampus in human temporal lobe epilepsy. Acta. Neuropathol.

[40] Lehmann GL, Gradilone SA, Marinelli RA (2004). Aquaporin water channels in central nervous system. Curr. Neurovasc. Res.

[41] Li X, Kong H, Wu W, Xiao M, Sun X, Hu G (2009). Aquaporin-4 maintains ependymal integrity in adult mice. Neuroscience.

[42] Li J, Patil RV, Verkman AS (2002). Mildly abnormal retinal function in transgenic mice without Müller cell aquaporin-4 water channels. Invest. Ophthalmol. Vis. Sci.

[43] Li J, Verkman AS (2001). Impaired hearing in mice lacking aquaporin-4 water channels. J. Biol. Chem.

[44] Longatti P, Basaldella L, Orvieto E, Dei Tos AP, Martinuzzi A (2006). Aquaporin 1 expression in cystic hemangioblastomas. Neurosci. Lett.

[45] Mangiardi JR, Yodice P (1990). Metabolism of the malignant astrocytoma. Neurosurgery.

[46] Manley GT, Binder DK, Papadopoulos MC, Verkman AS (2004). New insights into water transport and edema in the central nervous system from phenotype analysis of aquaporin-4 null mice. Neuroscience.

[47] Manley GT, Fujimura M, Ma T, Noshita N, Filiz F, Bollen AW, Chan P, Verkman AS (2000). Aquaporin-4 deletion in mice reduces brain edema after acute water intoxication and ischemic stroke. Nat. Med.

[48] Marcus DC, Wu T, Wangemann P, Kofuji P (2002). KCNJ10 (Kir4.1) potassium channel knockout abolishes endocochlear potential. Am. J. Physiol. Cell. Physiol.

[49] Masseguin C, Corcoran M, Carcenac C, Daunton NG, Güell A, Verkman AS, Gabrion J (2000). Altered gravity downregulates aquaporin-1 protein expression in choroid plexus. J. Appl. Physiol.

[50] Meng S, Qiao M, Lin L, Del Bigio MR, Tomanek B, Tuor UI (2004). Correspondence of AQP4 expression and hypoxic-ischaemic brain oedema monitored by magnetic resonance imaging in the immature and juvenile rat. Eur. J. Neurosci.

[51] Merves M, Bobbitt B, Parker K, Kishore BK, Choo D (2000). Developmental expression of aquaporin 2 in the mouse inner ear. Laryngoscope.

[52] Mobasheri A, Marples D (2004). Expression of the AQP-1 water channel in normal human tissues: a semiquantitative study using tissue microarray technology. Am. J. Physiol. Cell. Physiol.

[53] Mobasheri A, Wray S, Marples D (2005). Distribution of AQP2 and AQP3 water channels in human tissue microarrays. J. Mol. Histol.

[54] Nagahama M, Ma N, Semba R, Naruse S (2006). Aquaporin 1 immunoreactive enteric neurons in the rat ileum. Neurosci. Lett.

[55] Nagelhus EA, Mathiisen TM, Ottersen OP (2004). Aquaporin-4 in the central nervous system: cellular and subcellular distribution and coexpression with KIR4.1. Neuroscience.

[56] Nagelhus EA, Veruki ML, Torp R, Haug FM, Laake JH, Nielsen S, Agre P, Ottersen OP (1998). Aquaporin-4 water channel protein in the rat retina and optic nerve: polarized expression in Müller cells and fibrous astrocytes. J. Neurosci.

[57] Nandasena BG, Suzuki A, Aita M, Kawano Y, Nozawa-Inoue K, Maeda T (2007). Immunolocalization of aquaporin-1 in the mechanoreceptive Ruffini endings in the periodontal ligament. Brain Res.

[58] Nesic O, Lee J, Unabia GC, Johnson K, Ye Z, Vergara L, Hulsebosch CE, Perez-Polo JR (2008). Aquaporin 1 - a novel player in spinal cord injury. J. Neurochem.

[59] Nesic O, Lee J, Ye Z, Unabia GC, Rafati D, Hulsebosch CE, Perez-Polo JR (2006). Acute and chronic changes in aquaporin 4 expression after spinal cord injury. Neuroscience.

[60] Newman EA, Kettenmann H, Ransom BR (1995). Glial cell regulation of extracellular potassium. Neuroglia.

[61] Nicchia GP, Frigeri A, Nico B, Ribatti D, Svelto M (2001). Tissue distribution and membrane localization of aquaporin-9 water channel: evidence for sex-linked differences in liver. J. Histochem. Cytochem.

[62] Nicchia GP, Srinivas M, Li W, Brosnan CF, Frigeri A, Spray DC (2005). New possible roles for aquaporin-4 in astrocytes: cell cytoskeleton and functional relationship with connexin43. FASEB J.

[63] Nielsen S, Nagelhus EA, Amiry-Moghaddam M, Bourque C, Agre P, Ottersen OP (1997). Specialized membrane domains for water transport in glial cells: high-resolution immunogold cytochemistry of aquaporin-4 in rat brain. J. Neurosci.

[64] Oshio K, Binder DK, Liang Y, Bollen A, Feuerstein B, Berger MS, Manley GT (2005). Expression of the aquaporin-1 water channel in human glial tumors. Neurosurgery.

[65] Oshio K, Binder DK, Yang B, Schecter S, Verkman AS, Manley GT (2004). Expression of aquaporin water channels in mouse spinal cord. Neuroscience.

[66] Oshio K, Watanabe H, Song Y, Verkman AS, Manley GT (2005). Reduced cerebrospinal fluid production and intracranial pressure in mice lacking choroid plexus water channel Aquaporin-1. FASEB J.

[67] Oshio K, Watanabe H, Yan D, Verkman AS, Manley GT (2006). Impaired pain sensation in mice lacking Aquaporin-1 water channels. Biochem. Biophys. Res. Commun.

[68] Pan E, Stringer JL (1996). Influence of osmolality on seizure amplitude and propagation in the rat dentate gyrus. Neurosci. Lett.

[69] Papadopoulos MC, Manley GT, Krishna S, Verkman AS (2004). Aquaporin-4 facilitates reabsorption of excess fluid in vasogenic brain edema. FASEB J.

[70] Papadopoulos MC, Saadoun S, Binder DK, Manley GT, Krishna S, Verkman AS (2004). Molecular mechanisms of brain tumor edema. Neuroscience.

[71] Papadopoulos MC, Saadoun S, Verkman AS (2008). Aquaporins and cell migration. Pflugers Arch.

[72] Pardo CA, Vargas DL, Zimmerman AW (2005). Immunity, neuroglia and neuroinflammation in autism. Int. Rev. Psychiatry.

[73] Pérez E, Barrachina M, Rodríguez A, Torrejón-Escribano B, Boada M, Hernández I, Sánchez M, Ferrer I (2007). Aquaporin expression in the cerebral cortex is increased at early stages of Alzheimer disease. Brain Res.

[74] Preston GM, Carroll TP, Guggino WB, Agre P (1992). Appearance of water channels in Xenopus oocytes expressing red cell CHIP28 protein. Science.

[75] Quagliarello VJ, Long WJ, Scheld WM (1986). Morphologic alterations of the blood-brain barrier with experimental meningitis in the rat. Temporal sequence and role of encapsulation. J. Clin. Invest.

[76] Raivich G, Bohatschek M, Kloss CU, Werner A, Jones LL, Kreutzberg GW (1999). Neuroglial activation repertoire in the injured brain: graded response, molecular mechanisms and cues to physiological function. Brain Res. Brain Res. Rev.

[77] Rash JE, Yasumura T, Dudek FE, Nagy JI (2001). Cell-specific expression of connexins and evidence of restricted gap junctional coupling between glial cells and between neurons. J. Neurosci.

[78] Rash JE, Yasumura T, Hudson CS, Agre P, Nielsen S (1998). Direct immunogold labeling of aquaporin-4 in square arrays of astrocyte and ependymocyte plasma membranes in rat brain and spinal cord. Proc. Natl. Acad. Sci. USA.

[79] Rodríguez A, Pérez-Gracia E, Espinosa JC, Pumarola M, Torres JM, Ferrer I (2006). Increased expression of water channel aquaporin 1 and aquaporin 4 in Creutzfeldt-Jakob disease and in bovine spongiform encephalopathy-infected bovine-PrP transgenic mice. Acta. Neuropathol.

[80] Saadoun S, Papadopoulos M, Bell B, Krishna S, Davies D (2002). The aquaporin-4 water channel and brain tumour oedema. J. Anat.

[81] Saadoun S, Papadopoulos MC, Davies DC, Bell BA, Krishna S (2002). Increased aquaporin 1 water channel expression in human brain tumours. Br. J. Cancer.

[82] Satoh J, Tabunoki H, Yamamura T, Arima K, Konno H (2007). Human astrocytes express aquaporin-1 and aquaporin-4 *in vitro* and *in vivo*. Neuropathology.

[83] Schwartzkroin PA, Baraban SC, Hochman DW (1998). Osmolarity, ionic flux, and changes in brain excitability. Epilepsy Res.

[84] Shields SD, Mazario J, Skinner K, Basbaum AI (2007). Anatomical and functional analysis of aquaporin 1, a water channel in primary afferent neurons. Pain.

[85] Simard M, Nedergaard M (2004). The neurobiology of glia in the context of water and ion homeostasis. Neuroscience.

[86] Solenov EI, Vetrivel L, Oshio K, Manley GT, Verkman AS (2002). Optical measurement of swelling and water transport in spinal cord slices from aquaporin null mice. J. Neurosci. Methods.

[87] Speake T, Freeman LJ, Brown PD (2003). Expression of aquaporin 1 and aquaporin 4 water channels in rat choroid plexus. Biochim. Biophys. Acta.

[88] St. Hillaire C, Vargas D, Pardo CA, Gincel D, Mann J, Rothstein JD, McArthur JC, Conant K (2005). Aquaporin 4 is increased in association with human immunodeficiency virus dementia: implications for disease pathogenesis. J. Neurovirol.

[89] Stevens AL, Breton S, Gustafson CE, Bouley R, Nelson RD, Kohan DE, Brown D (2000). Aquaporin 2 is a vasopressin-independent, constitutive apical membrane protein in rat vas deferens. Am. J. Physiol. Cell. Physiol.

[90] Streit WJ, Walter SA, Pennell NA (1999). Reactive microgliosis. Prog. Neurobiol.

[91] Sun MC, Honey CR, Berk C, Wong NL, Tsui JK (2003). Regulation of aquaporin-4 in a traumatic brain injury model in rats. J. Neurosurg.

[92] Suzuki R, Okuda M, Asai J, Nagashima G, Itokawa H, Matsunaga A, Fujimoto T, Suzuki T (2006). Astrocytes co-express aquaporin-1, -4, and vascular endothelial growth factor in brain edema tissue associated with brain contusion. Acta Neurochir. Suppl.

[93] Tait MJ, Saadoun S, Bell BA, Papadopoulos MC (2008). Water movements in the brain: role of aquaporins. Trends Neurosci.

[94] Tan G, Sun SQ, Yuan DL (2008). Expression of the water channel protein aquaporin-9 in human astrocytic tumours: correlation with pathological grade. J. Int. Med. Res.

[95] Tomás-Camardiel M, Venero JL, de Pablos RM, Rite I, Machado A, Cano J (2004). *In vivo* expression of aquaporin-4 by reactive microglia. J. Neurochem.

[96] Tsukaguchi H, Shayakul C, Berger UV, Mackenzie B, Devidas S, Guggino WB, van Hoek AN, Hediger MA (1998). Molecular characterization of a broad selectivity neutral solute channel. J. Biol. Chem.

[97] Umenishi F, Schrier RW (2003). Hypertonicity-induced aquaporin-1 (AQP1) expression is mediated by the activation of MAPK pathways and hypertonicity-responsive element in the AQP1 gene. J. Biol. Chem.

[98] Vargas DL, Nascimbene C, Krishnan C, Zimmerman AW, Pardo CA (2005). Neuroglial activation and neuroinflammation in the brain of patients with autism. Ann. Neurol.

[99] Verkman AS (2006). Roles of aquaporins in kidney revealed by transgenic mice. Semin. Nephrol.

[100] Walz W (1987). Swelling and potassium uptake in cultured astrocytes. Can. J. Physiol. Pharmacol.

[101] Walz W (1992). Mechanism of rapid K(^+^)-induced swelling of mouse astrocytes. Neurosci. Lett.

[102] Warth A, Kröger S, Wolburg H (2004). Redistribution of aquaporin-4 in human glioblastoma correlates with loss of agrin immunoreactivity from brain capillary basal laminae. Acta Neuropathol.

[103] Warth A, Mittelbronn M, Hülper P, Erdlenbruch B, Wolburg H (2007). Expression of the water channel protein aquaporin-9 in malignant brain tumors. Appl. Immunohistochem. Mol. Morphol.

[104] Warth A, Mittelbronn M, Wolburg H (2005). Redistribution of the water channel protein aquaporin-4 and the K^+^ channel protein Kir4.1 differs in low- and high-grade human brain tumors. Acta Neuropathol.

[105] Warth A, Simon P, Capper D, Goeppert B, Tabatabai G, Herzog H, Dietz K, Stubenvoll F, Ajaaj R, Becker R, Weller M, Meyermann R, Wolburg H, Mittelbronn M (2007). Expression pattern of the water channel aquaporin-4 in human gliomas is associated with blood-brain barrier disturbance but not with patient survival. J. Neurosci. Res.

[106] Xu Q, Wang S, Jiang X, Zhao Y, Gao M, Zhang Y, Wang X, Tano K, Kanehara M, Zhang W, Ishida T (2007). Hypoxia-induced astrocytes promote the migration of neural progenitor cells *via* vascular endothelial factor, stem cell factor, stromal-derived factor-1alpha and monocyte chemoattractant protein-1 upregulation *in vitro*. Clin. Exp. Pharmacol. Physiol.

[107] Yamamoto N, Sobue K, Fujita M, Katsuya H, Asai K (2002). Differential regulation of aquaporin-5 and -9 expression in astrocytes by protein kinase A. Brain Res. Mol. Brain Res.

[108] Yamamoto N, Yoneda K, Asai K, Sobue K, Tada T, Fujita Y, Katsuya H, Fujita M, Aihara N, Mase M, Yamada K, Miura Y, Kato T (2001). Alterations in the expression of the AQP family in cultured rat astrocytes during hypoxia and reoxygenation. Brain Res. Mol. Brain Res.

[109] Zeidel ML, Ambudkar SV, Smith BL, Agre P (1992). Reconstitution of functional water channels in liposomes containing purified red cell CHIP28 protein. Biochemistry.

[110] Zhang D, Vetrivel L, Verkman AS (2002). Aquaporin deletion in mice reduces intraocular pressure and aqueous fluid production. J. Gen. Physiol.

[111] Zhao J, Moore AN, Clifton GL, Dash PK (2005). Sulforaphane enhances Aquaporin-4 expression and decreases cerebral edema following traumatic brain injury. J. Neurosci. Res.

